# Advances of exosomes in periodontitis treatment

**DOI:** 10.1186/s12967-022-03487-4

**Published:** 2022-06-21

**Authors:** Hongbing Lin, Huishan Chen, Xuetao Zhao, Tong Ding, Yawei Wang, Zhen Chen, Yue Tian, Peipei Zhang, Yuqin Shen

**Affiliations:** 1 Jilin Provincial Key Laboratory of Tooth Development and Bone Remodeling, Hospital of Stomatology, Jilin University, Changchun, Jilin 130021 People’s Republic of China; 2 Department of Periodontics, Affiliated Stomatology Hospital of Guangzhou Medical University,, Guangzhou Key Laboratory of Basic and Applied Research of Oral Regenerative Medicine, Guangzhou, Guangdong 510182 People’s Republic of China

**Keywords:** Periodontitis, Exosomes, Immunomodulation, Immune cells, Osteoclast

## Abstract

Periodontitis is an inflammatory disease initiated by dysbiosis of the local microbial community. Periodontitis can result in destruction of tooth-supporting tissue; however, overactivation of the host immune response is the main reason for alveolar bone loss. Periodontal tissue cells, immune cells, and even further activated osteoclasts and neutrophils play pro-inflammatory or anti-inflammatory roles. Traditional therapies for periodontitis are effective in reducing the microbial quantities and improving the clinical symptoms of periodontitis. However, these methods are non-selective, and it is still challenging to achieve an ideal treatment effect in clinics using the currently available treatments and approaches. Exosomes have shown promising potential in various preclinical and clinical studies, including in the diagnosis and treatment of periodontitis. Exos can be secreted by almost all types of cells, containing specific substances of cells: RNA, free fatty acids, proteins, surface receptors and cytokines. Exos act as local and systemic intercellular communication medium, play significant roles in various biological functions, and regulate physiological and pathological processes in numerous diseases. Exos-based periodontitis diagnosis and treatment strategies have been reported to obtain the potential to overcome the drawbacks of traditional therapies. This review focuses on the accumulating evidence from the last 5 years, indicating the therapeutic potential of the Exos in preclinical and clinical studies of periodontitis. Recent advances on Exos-based periodontitis diagnosis and treatment strategies, existing challenges, and prospect are summarized as guidance to improve the effectiveness of Exos on periodontitis in clinics.

## Background

**Fig. 1:**
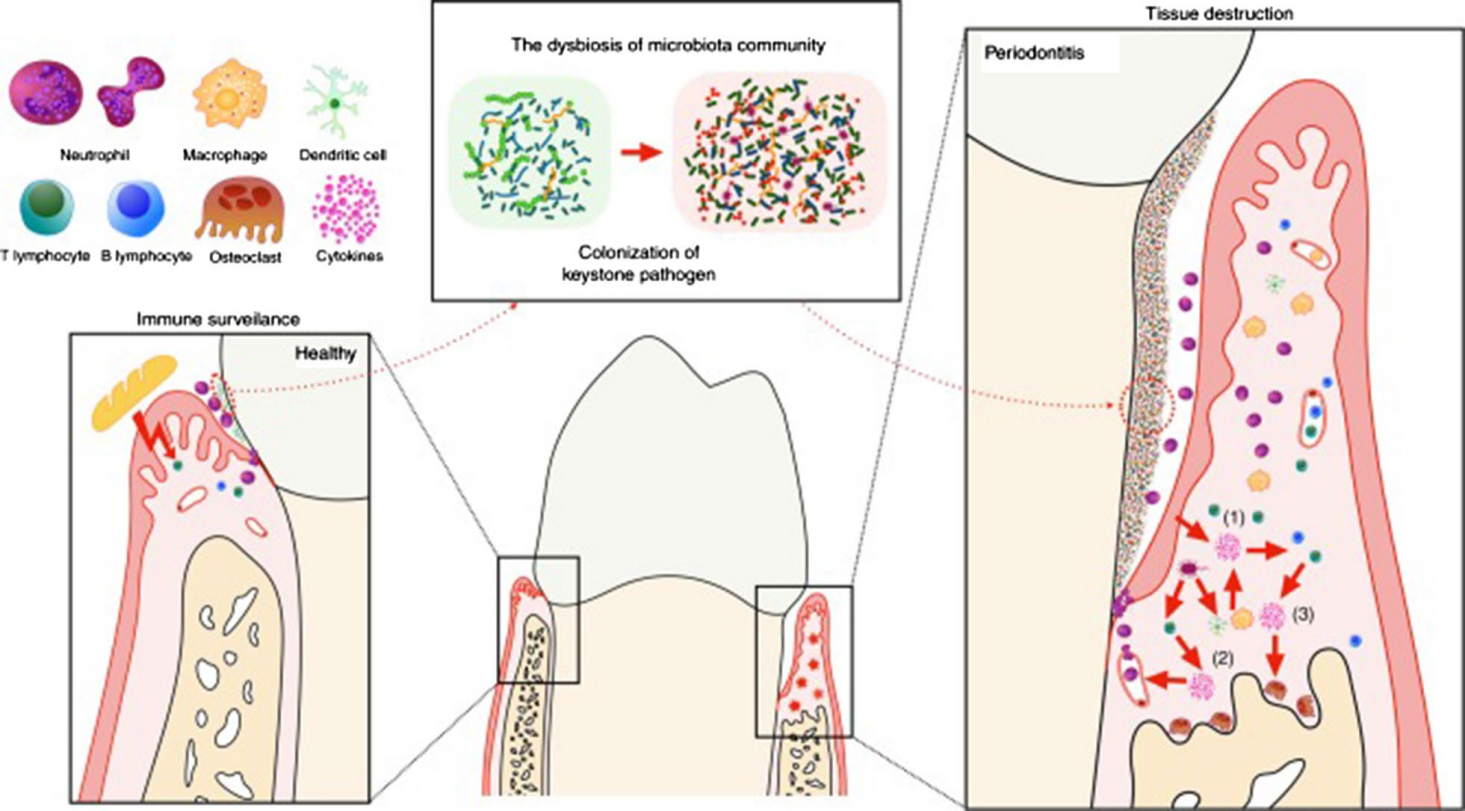
The host cells involved in the process of periodontitis. The dysbiosis of the local microbial over-activate the host immune response, the interaction between the microbiota and all host cells leads eventually leads to tissue destruction [4]. Reprinted with permission. Copyright (2019), Springer Nature

Periodontitis is an inflammatory disease initiated by dysbiosis of the local microbial community and is characterized by the relative abundance or influence imbalance of microbial species [1]. However, overactivation of the host immune response is the main reason for the direct activation of osteoclast activity and alveolar bone loss [2]. The composition and total number of microbiota change after the colonization of "keystone" pathogen, which improves the pathogenicity of the whole community. Thus, the immune response is overactivated, resulting in immune cells infiltration, activation of osteoclast activity, and destruction of soft and hard tissues [3]. Pathological immunity of the host to dysbiotic microbes first occurs between the microbiome and host cells, which include periodontal tissue cells and other immune cells, such as mononuclear phagocytes (MNPs), antigen-presenting cells (APCs), and specific T cell subsets. Naive T cells and B cells not only differentiate into mature T cells or plasma cells but also further activate or promote osteoclasts and neutrophils to play a pro-inflammatory or anti-inflammatory role [4] (Fig. 1).

Traditional therapies for periodontitis include scaling and root planning (SRP), systemic and local administration of antibiotics, and oral antiseptics. In the short term, these therapies are usually effective in reducing the microbial quantities in the tissues and blood of periodontitis patients and improving the clinical symptoms of periodontitis. However, these methods are non-selective, and the oral and systemic effects of long-term medication need to be evaluated [5]. It is becoming increasingly clear that new strategies need to be developed to treat periodontitis more effectively.

Exos were discovered in 1987 and have been shown to be important in cell communication [6]. Other studies have confirmed that Exos are a natural nanoparticle delivery method that can treat multiple infectious and immune/inflammatory diseases [7–10]. Thus, in this review, we summarized studies from the last 5 years that have focused on the effect of Exos on periodontitis and host cells.

## Exos

### The biogenesis of Exos

Exos are secreted by almost all types of cells, including mesenchymal stem cells (MSCs), dendritic cells (DCs), B cells, T cells, and mast cells, and widely exist in many body fluids such as plasma, urine, breast milk, semen, amniotic fluid, and saliva [11]. Exos are among the three major types of extracellular vesicles (EVs). The size of the Exos is between 50 and 150 nm and the density is between 1.15 and 1.19 g / ml [12]. The biogenesis and release of Exos are different from other of other microbubbles. While other microbubbles are released directly through the plasma membrane, Exos originating from endocytosis [13]. First, the membrane of secretory cells is sunken inward to produce endocytic vesicles. Multiple endocytic vesicles combine to form early nucleosomes, and then miRNA, mRNA and DNA are packaged in the cytoplasm to form late endocytosis vesicles. The late endocytic vesicles germinate inward to form intracavitary vesicles (ILVs). The aggregation of ILVs in late endosomes create multivesicular bodies (MVBs), which are formed by the inward invagination of the endosomal limiting membrane. Finally, some MVBs with low cholesterol were degraded by lysosomes, and some MVBs, rich in cholesterol, release extracellular bodies through the fusion of the cell membrane with itself [13–16]. The active formation of Exos is dependent on the endosomal sorting complex required for transport (ESCRT; ESCRT-0, I, II, III and Vps4) and its accessory proteins (Alix, TSG101, HSC70, and HSP90β). They recognize ubiquitinated transmembrane proteins and incorporate endosomal proteins into MVBs. [17, 18]. Passive formation of Exos is independent of ESCRT and involves lipids (ceramide), tetrapeptides (CD63) and heat shock proteins which induce cell membrane budding and promote MVB formation [19, 20]. In addition, certain components, such as four-transmembrane domain proteins and lipid rafts, have been reported to participate in the formation of some exosomes [21, 22]. In addition to the classic pathway, there is a much more immediate route of exosome biogenesis. T cells and erythroleukemia cell lines can release exosomes from the plasma membrane directly, and the Exos produced by these two pathways cannot be distinguished [23, 24]. Furthermore, soluble N-ethylmaleimide-sensitive fusion protein attachment protein receptor (SNARE) proteins and their effectors such as Rab GTPases (Rab27a, Rab27b and Rab35) play a significant role in exosome secretion [14].

**Fig. 2:**
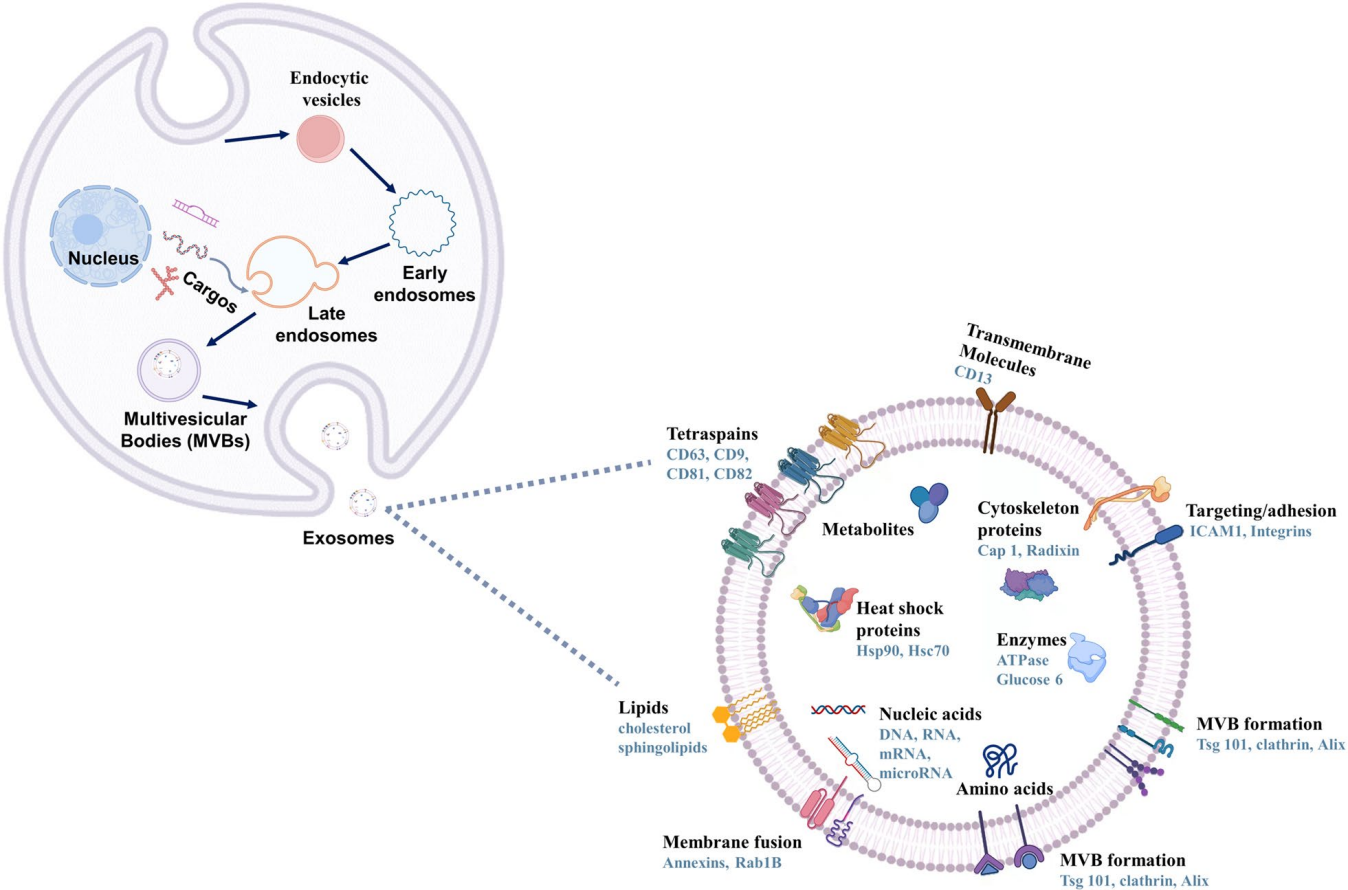
The biogenesis, formation, and content of Exos

The biogenesis of Exos is affected by many external factors, including cell type, serum conditions, cytokines, and growth factors. The heterogeneity of Exos is based on their specific morphology, content, and function [25, 26]. Encapsulated by lipid membranes derived from parental cells, Exos show inherent histocompatibility and tissue orientation mediated by surface molecules such as integrin and glycans [27]. In addition, Exos contain four transmembrane proteins (CD9, CD63, CD81 and CD82), heat shock proteins, lipoproteins, and some transport-related proteins. These proteins can not only provide markers for Exos identification but also locate Exos in specific target cells [28, 29]. Exos contain substances to mother cells, such as RNA, free fatty acids, proteins, surface receptors, and cytokines, which act as local and systemic intercellular communication media [30, 31]. Owing to these characteristics, Exos play a significant role in various biological functions and regulate many physiological and pathological processes in numerous diseases [32] (Fig. 2).

### The isolation and characterization of Exos

**Fig. 3:**
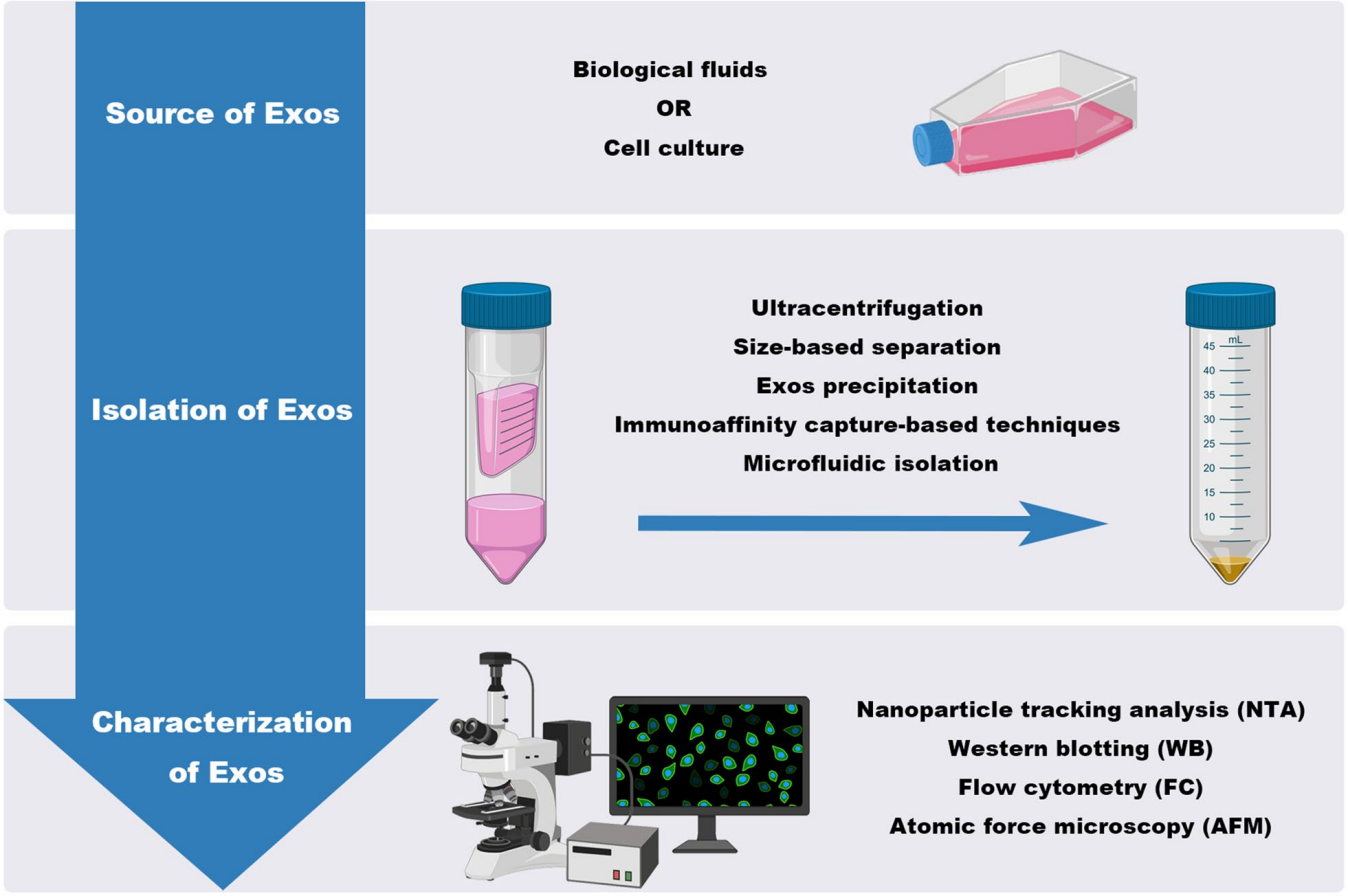
A schematic diagram depicting the isolation and characterization of Exos

The selection and improvement of isolation strategies should be determined according to the kind of biological fluids Exos are isolated from. Effective isolation strategies should be able to concentrate the signals of the Exos to be analyzed and avoid contamination with other molecules, such as lipoproteins, non-vesicular protein aggregates, and other EVs, which are similar to Exos in terms of size and density. At present, there are many techniques for separating and purifying Exos from biological fluids and in vitro cell cultures, including ultracentrifugation, size-based separation, Exos precipitation, immunoaffinity capture-based techniques, and microfluidic isolation [33–36]. Among these, differential centrifugation is the most widely used and basic method for Exos separation, and it is a feasible strategy to combine two or more methods to improve the yield, purity, and efficiency of Exos extracrion. Various techniques based on biophysical or biological characteristics have been used to verify upstream Exos separation methods and classify Exos subgroups for downstream analysis, such as nanoparticle tracking analysis (NTA), western blotting (WB), flow cytometry (FC), and atomic force microscopy (AFM) [35–37] (Fig. 3).

## Exos based periodontitis diagnostic and treatment strategy

Periodontitis is a major public health problem with a high incidence rate worldwide. It could not only cause destruction of the supporting tissues of teeth but also have a negative effect on systematic disease states [38]. Therefore, effective diagnosis and treatment of periodontitis are important to reduce the risk of periodontitis.

### Exos-based periodontitis diagnostic strategy

Because the components of Exos can be reprogrammed according to disease status, Exos are increasingly being evaluated as potential diagnostic biomarkers for the diagnosis and prognosis of diseases. These characteristics have made Exos a focus of oral disease research in recent years, including periodontitis [39].

At the gene level, Exos are enriched in specific microRNAs (miRNAs), that can provide disease-specific diagnostic signatures [40]. When compared to healthy controls, plasma-derived exosomal miRs (miR-1304-3p and miR-200c-3p) and snoRs (SNORD57 and SNODB1771) from periodontitis patients are differentially expressed and could be the valuable biomarkers for periodontitis diagnosis [41]. In addition, the level of programmed death-ligand 1 (PD-L1) mRNA in salivary Exos may have the potential to diagnose periodontitis and is relative to the severity of periodontitis [42].

At the protein level, detection and analysis of salivary exosomal proteins in young adults with severe periodontitis (SP) suggested that C6 proteins, which participate in the immune response during the development of periodontitis, were expressed only in the SP group [43].

In addition, levels of CD9 and CD81 Exos in periodontitis patients were significantly lower than those in the healthy controls. Because the concentration of CD9/CD81 Exos in saliva is significantly and negatively correlated with clinical measurements, it may be of great significance in the pathogenesis of periodontal disease [44]. Other advances in salivary Exos in the diagnosis of periodontitis are well-reviewed in the literature [45].

### Exos-based periodontitis treatment strategy

Exos have been reported to provide a novel perspective and potential therapeutic approach for treating periodontitis and improving alveolar resorption. Exos derived from 3D-cultured MSCs restored not only the Th17 cell/Treg balance through the miR-1246/Nfat5 axis, but also the immune responses in the inflamed periodontium [46]. Dental pulp stem cell-Exo (DPSC-Exos) can facilitate the conversion of macrophages from a pro-inflammatory phenotype (M1)to an anti-inflammatory phenotype (M2) and promote the healing of alveolar bone in mice with periodontitis, the mechanism of which could be associated with miR-1246 in DPSC-Exos [47]. Exos purified from human leukocyte antigen haplotype homo dental pulp cell lines (HHH-DPCs) stimulated the migration of human DPCs and mouse osteoblastic and significantly suppressed osteoclast formation in vitro [48]. Exos secreted from healthy periodontal ligament stem cells (PDLSCs) promote osteogenic differentiation of PDLSCs derived from periodontitis tissue. Healthy PDLSC-Exos (h-PDLSC-Exos) treatment resulted in accelerated bone formation in alveolar bone defects in rat models of periodontitis. Mechanistically, h-PDLSC-Exos suppressed the overactivation of canonical Wnt signaling to recover the osteogenic differentiation capacity of inflammatory PDLSCs [49]. Exos derived from TNF-α-preconditioned gingival mesenchymal stem cells (GMSCs) could significantly regulate inflammation and osteoclastogenesis, which could provide a therapeutic approach for periodontitis [50]. Human exfoliated deciduous teeth (SHED)-derived Exos (SHED-Exos) restored bone loss in mouse periodontitis model and promoted bone marrow stromal cells (BMSCs) osteogenesis, differentiation, and bone formation [51]. SHED-Exos contribute to periodontal bone regeneration by promoting neovascularization and new bone formation, possibly through the AMPK signaling pathway [52]. Exos from reparative M2 macrophages reduced alveolar bone resorption in mice with periodontitis via the IL-10/IL-10R pathway [53]. Exos derived from adipose-derived stem cells (ADSC-Exos) represent a promising adjunctive treatment to SRP in rats [54]. Exosomal miR-25-3p in saliva contributes to the development and progression of diabetes-associated periodontitis. The discovery of other miR-25-3p targets may provide critical insights into the development of drugs to treat periodontitis by regulating γδ T cell-mediated local inflammation [55]. The in vivo effects of Exos on periodontitis are summarized in Table 1.

**Table 1:** Summary of in vivo results showing the Exos-based periodontitis treatment strategy used over the past 5 years

Source of Exos	Study model	Route of delivery	Dose	Duration	Outcomes	References
MSCs	Mouse experimental periodontitis model	Locally injection	50 μg per mouse	14 days	Improve the treatment by restoring the Th17 cell/Treg balance through the miR-1246/Nfat5 axis	[46]
DPSCs	Mouse experimental periodontitis model	Incorporated chitosan hydrogel	50 μg	4 weeks	Facilitate macrophages from M1 to M2 phenotype and promote alveolar bone healing	[47]
HHH-DPSCs	Mouse experimental periodontitis model	Directly applied onto the silk ligature	5 μL containing 7.5 × 10^8^ particles	7 days	Promote the migration of both DPCs and osteoblastic cells; suppress osteoclast formation	[48]
PDLSCs	Rat periodontal bone defect model	Mixed with Matrigel	Exos (225 μg/μL): Matrigel = 2:1 (v/v)	4 weeks	Suppress overactivation Wnt signaling, recover osteogenic differentiation capacity of inflammatory PDLSCs	[49]
TNF-α-treated human GMSCs	Mouse experimental periodontitis model	Locally injection	20 μg per mouse	7 days	Regulate inflammation and osteoclastogenesis	[50]
SHED	Mouse experimental periodontitis model	Locally injection	20 μg	2 weeks	Restore bone loss, promote BMSCs osteogenesis, differentiation, and bone formation	[51]
SHED	Rat periodontal defect models	h β-TCP scaffffolds loaded with Exos	2 μg/μL Exos in 100 μL PBS	4 weeks	Contribute to periodontal bone regeneration through the AMPK signaling pathway	[52]
induced M2-like macrophages	Mouse experimental periodontitis model	Locally injection	30 μL (500 ng/ml)	2 weeks	Reduce alveolar bone resorption in mice with periodontitis via IL-10/IL-10R pathway	[53]
ADSCs	Rat experimental periodontitis model	Locally injection	80–150 µg in 200 µL PBS	4 weeks	Represent a promising adjunctive treatment to SRP	[54]
salivary Exos	Insulin resistance-associated mouse experimental periodontitis model	Locally injection	miR-25-3p inhibitors (100 μl of 8 nM)	9 days	Exosomal miR-25-3p in saliva contribute to development and progression of diabetes-associated periodontitis	[55]

## Possible mechanism of Exos on host cells during periodontitis

**Fig. 4:**
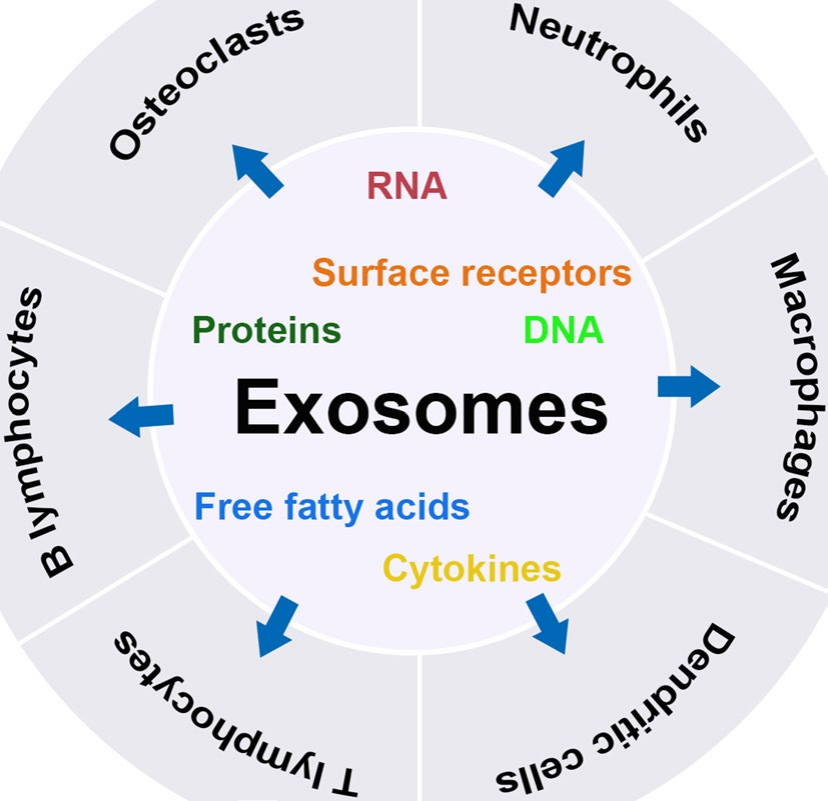
Biological activities of Exos modifying the host cells during periodontitis

Overactivation of the host immune response is caused by the interaction between the dysbiosis of local microbes and host cells, eventually leading to periodontal tissue destruction. The host cells include periodontal tissue cells and other immune cells, which play pro-inflammatory or anti-inflammatory roles [4]. Exos retain proteins, miRNA, mRNA, DNA, and lipids, and can transfer that cargo to distant target cells and modify the target cells. Reports have revealed the biological activities of Exos in modifying host cells (Fig. 4). Modification of host cells with Exos from different sources plays an important role in the treatment of periodontitis.

### Effects of Exos on neutrophils

Neutrophils are short-lived cells in the innate immune system. They play an important role in pathogen resistance by producing reactive oxygen species (ROS). Therefore, effective strategies to improve the viability and function of neutrophils may be beneficial for treating infections and immune deficiency diseases. MSC-Exos have a protective effect on neutrophil function and lifespan [56], and could significantly reduce the terminal complement activation complex C5b-9 to inhibit neutrophils accumulation [57]. Exos isolated from ADSCs (ADSC-Exos) can decrease neutrophil apoptosis and increase phagocytosis [58]. Exos isolated from LPS-treated macrophages can induce cytokine production and neutrophils migration [59].

### Effects of Exos on macrophages

MSC-Exos can modify the polarization of the pro-inflammatory phenotype (M1 macrophages) to the anti-inflammatory phenotype (M2 macrophages) via shuttling miR-182 [60]. Exos derived from BMSCs (BMSC-Exos) can increase M2 macrophages [61], and BMSC-Exos have been reported to inhibit M1 macrophages and promote M2 macrophages in a murine alveolar macrophage cell line by inhibiting cellular glycolysis [62]. FNDC5 pre-conditioned BMSC-Exos have also been confirmed to play an anti-inflammatory role and promote M2 macrophages via NF-κB signaling pathway and the Nrf2/HO-1 axis [63]. Exos from human umbilical cord mesenchymal stem cells (hUCMSC-Exos) facilitated CD163 + M2 macrophages [64] and promoted M2 macrophages in LPS-stimulated RAW 264.7 via tumor necrosis factor receptor-associated factor 1 (TRAF1) [65]. ADSC-Exos can significantly upregulate the mRNA expression of M2 macrophages [66], induce M2 macrophages through the transactivation of Arg-1 by Exos-carried active STAT3 [67], and alleviate LPS induced inflammation by regulating Nrf2/HO-1 expression (68). Exos from GMSCs (GMSC-Exos) and dental pulp stem cells (DPSC-Exos) can promote the transformation of macrophages from M1 to M2 [47, 69]. TNF-α stimulated GMSC-Exos have also been reported to induce anti-inflammatory M2 macrophage polarization [47, 50].

### Effects of Exos on DCs

MSC-Exos decreased DC surface marker expression in cells treated with LPS and decreased lymphocyte proliferation in the presence of MSC-Exos treated DCs, suggesting that MSC-Exos may play a key role in DC-induced immune responses [70]. hUCMSC-Exos suppressed the maturation and activation of DC and decreased the expression of IL-23, which is particularly important for promoting the pathogenicity of Th17 cells [71]. Exos from RegDC (RegDC-Exos) suppress the maturation of DCs and promote the recruitment of Treg cells, resulting in the inhibition of bone resorptive cytokines and reduction in osteoclastic bone loss [72]. Exos from lymphatic endothelial cells (LEC-Exos) promote the directional migration of human DCs in complex tissue environments in a CX3CL1/fractalkine-dependent fashion [73].

### Effects of Exos on T lymphocytes

MSC-Exos decreased T lymphocyte proliferation and the percentage of CD4 + and CD8 + T cell subsets in a dose-dependent manner while increasing Treg cell populations [74]. MSC-Exos promote the proliferation and immune-suppression capacity of Tregs by upregulating IL-10 and TGF-β1[75], and inhibit the differentiation of Th2 cells by regulating the miR-146a-5p/SERPINB2 pathway [76]. PDLSC-Exos alleviated inflammatory microenvironment and maintained Th17/Treg balance via the Th17/Treg/miR‐155‐5p/SIRT1 regulatory network [77]. CD137-modified endothelial cell-Exos (EC-Exos) promote Th17 cell differentiation via the NF-КB pathway by regulating IL-6 expression [78].

### Effects of Exos on B lymphocytes

MSC-Exos had the beneficial effect of reducing plasmablasts and incresing Breg-like cells in lymph nodes [74].

### Effects of Exos on osteoclasts

Exosomal miR-1260b of TNF-α-preconditioned GMSC-Exos was found to inhibit osteoclastogenic activity by targeting the Wnt5a-mediated RANKL pathway [50]. RegDC-Exos inhibit the production of bone resorptive cytokines and bone loss in osteoclasts [72]. Cyclic mechanical stretch (CMS)-treated BMSC-Exos can impair osteoclast differentiation by inhibiting the RANKL-NF-κB signaling pathway [79]. ADSC-Exos can reduce bone resorption and recover bone loss by suppressing NLRP3 inflammasome activation in osteoclasts [80] and by antagonizing osteocyte-mediated osteoclastogenesis [81]. ADSC-Exos combined with microRNA-146a (miR-146a-Exo) were reported to restrain bone resorption by inhibiting pro-inflammatory cytokine production in high glucose-treated osteoclasts [82]. Exos derived from osteoblasts can inhibit osteoclast differentiation via the miR-503-3p/Hpse axis [83]. Exos from endothelial progenitor cells (EPC-Exos) can promote bone repair by enhancing the recruitment and differentiation of osteoclast precursors via LncRNA-MALAT1 [84].

The effects of Exos on the host cells involved in periodontitis are summarized in Table 2.

**Table 2:** Summary of the effects of Exos on host cells

No	Source of Exos	Biological activity	References
Neutrophil			
1	MSCs	Have protective effects on neutrophil function and lifespan	[56]
2	MSCs	Reduce terminal complement activation complex C5b-9 to inhibit neutrophils accumulation	[57]
3	ADSCs	Decrease neutrophils apoptosis and increased their phagocytosis capacity	[58]
4	LPS-treated macrophages	Induce cytokine production and neutrophil migration	[59]
Macrophage			
1	DPSCs	Facilitate macrophages to convert from M1 phenotype to M2 phenotype	[47]
2	TNF-α induced GMSCs	Induce anti-inflammatory M2 macrophage polarization	[50]
3	MSCs	Modify the polarization of M1 macrophages to M2 macrophages via shuttling miR-182	[60]
4	BMSCs	Increase M2 macrophage polarization	[61]
5	BMSCs	Inhibit M1 polarization and promotes M2 polarization in a murine alveolar macrophage cell line by inhibiting cellular glycolysis	[62]
6	FNDC5 pre-conditioned BMSCs	Play anti-inflammation effects and promote M2 macrophage polarization via NF-κB signaling pathway and Nrf2/HO-1 axis	[63]
7	hUCMSCs	Facilitate CD163 + M2 macrophage polarization, reduced inflammation, and increases anti-inflammatory responses	[64]
8	hUCMSCs	Inhibit M1 polarization and promoted M2 polarization through tumor necrosis factor receptor-associated factor 1 (TRAF1)	[65]
9	ADSCs	Upregulate mRNA expression of M2 macrophages	[66]
10	ADSCs	Induce anti-inflammatory M2 phenotypes through the transactivation of arginase-1 by Exo-carried active STAT3	[67]
11	ADSCs	Polarize macrophage to an anti-inflammatory phenotype via regulating the Nrf2/HO-1 expression	[68]
12	GMSCs	Facilitate macrophages to convert from M1 phenotype to M2 phenotype	[69]
Dendritic cell			
1	MSCs	Decrease DC surface marker expression and modulates DC-induced immune responses	[70]
2	hUCMSCs	Suppress maturation and activation of DCs, and decreases the expression level of IL-23	[71]
3	regDCs	Suppress maturation of recipient DCs resulting in inhibition of bone resorptive cytokines	[72]
4	LECs	Promote the directional migratory in a CX3CL1/fractalkine-dependent fashion	[73]
T lymphocyte			
1	MSCs	Increase Treg cell populations, inhibit T lymphocyte proliferation in a dose-dependent manner and decreases the percentage of CD4 + and CD8 + T cell subsets	[74]
2	MSCs	Upregulate IL-10 and TGF-β1 to promote proliferation and immune-suppression capacity of Tregs	[75]
3	MSCs	Inhibit the differentiation of Th2 cells via the regulation of the miR-146a-5p/SERPINB2 pathway	[76]
4	PDLSCs	Alleviate inflammatory microenvironment and keep Th17/Treg balance via Th17/Treg/miR‐155‐5p/SIRT1 regulatory network	[77]
5	CD137-modified ECs	Promote Th17 cell differentiation via NF-КB pathway mediated IL-6 expression	[78]
B lymphocyte			
1	MSCs	Upregulate Breg-like cells in lymph nodes	[74]
Osteoclast			
1	TNF-α-preconditioned GMSCs	Inhibit osteoclastogenic activity via exosomal miR-1260b to target Wnt5a-mediated RANKL pathway and	[50]
2	regDC	Result in inhibition of bone resorptive cytokines and reduces in osteoclastic bone loss	[72]
3	CMS-treated BMSCs	Impair osteoclast differentiation via inhibiting the RANKL-induced nuclear factor kappa-B (NF-κB) signaling pathway	[79]
4	ADSCs	Suppress NLRP3 inflammasome activation in osteoclasts and reduces bone resorption and recover bone loss	[80]
5	ADSCs	Antagonize osteocyte-mediated osteoclastogenesis	[81]
6	ADSCs	Inhibit pro-inflammatory cytokines production in high glucose-treated osteoclasts and restrains bone resorption	[82]
7	osteoblast	Inhibit the osteoclast differentiation via miR-503-3p/Hpse axis	[83]
8	EPCs	Promote bone repair by enhancing recruitment and differentiation of osteoclast precursors through LncRNA-MALAT1	(84)

## Summary and prospects

Exos can be secreted by almost all cell types and are the main contributor to cells efficacy. They are natural carriers of functional small RNA and proteins [85], and the constituents can be reprogrammed depending on the disease state [39]. Therefore, potential applications of Exos in the diagnosis and treatment of diseases are becoming increasingly popular. Exos derived from MSCs, with or without biomaterials, have broad application prospects in the treatment of periodontitis, especially in the cell-free treatment of tissue regeneration. Among them, Exos derived from oral stem cells are easier to collect and may show excellent characteristics of immune regulation, repair, and regeneration as well as less ethical, moral, or safety limits [12, 86]. In this review, we summarized the novel strategies using Exos in periodontitis over the last 5 years and analyze the possible mechanism of Exos in the treatment of periodontitis by summarizing the effect of Exos on host cells involved in the process of periodontitis.

Although the applications of Exos in periodontitis has been proved to be useful in animal models of preclinical research, much work needs to be done to apply it to clinics. Originally, Exos in clinical trials had to comply with good manufacturing practice (GMP), which includes the upstream of the cell culture process, the downstream of the purification process, and the quality control of Exos. The content carried by Exos varies from cell type to culture conditions and batch, which causes differences in biological functions. Therefore, it is necessary to explore more convenient and efficient technologies for the separation, purification, and storage of Exos to improve their homogeneity, purity, and repeatability. Furthermore, the corresponding role and mechanism of Exos in the diagnosis and treatment of periodontitis need to be explored more comprehensively. The critical range of differential expression of exosomes in periodontal tissue under healthy and inflammatory conditions for diagnosis and the amount and duration of safe and effective treatment need to be defined. Ultimately, the mechanisms of interaction between Exos and host cells are not clear, which makes it impossible for Exos to accurately regulate the target cells and functions. However, there is no doubt that Exos have the potential to provide personalized medical strategies for the prevention and treatment of periodontitis.

## Conclusions

Exos contain specific substances in their cells and play a significant role in the diagnosis and treatment of numerous diseases, including periodontitis. Exos-based periodontitis treatment strategies have been reported to obtain the potential to overcome the drawbacks of traditional therapies and have tremendous prospect for bench-to-bed translation.

## Data Availability

Not applicable.

## Abbreviations

MNPs: mononuclear phagocytes
APCs: antigen-presenting cells
SRP: scaling and root planning
MSCs: mesenchymal stem cells
DCs: dendritic cells
EVs: extracellular vesicles
SNARE: soluble N-ethylmaleimide-sensitive fusion protein attachment protein receptor
ILVs: intracavitary vesicles
MVBs: multivesicular bodies
NTA: nanoparticle tracking analysis
WB: western blotting
FC: flow cytometry
AFM: atomic force microscopy
miRNAs: microRNAs
PD-L1: programmed death-ligand 1
SP: severe periodontitis
DPSCs: dental pulp stem cells
HHH-DPCs: haplotype homo dental pulp cell lines
PDLSCs: periodontal ligament stem cells
GMSCs: gingival mesenchymal stem cells
SHED: human exfoliated deciduous teeth
BMSCs: bone marrow stromal cells
ADSCs: adipose-derived stem cells
ROS: reactive oxygen species
hUCMSCs: human umbilical cord mesenchymal stem cells
TRAF1: tumor necrosis factor receptor-associated factor 1
LECs: lymphatic endothelial cells
ECs: endothelial cells
CMS: cyclic mechanical stretch
EPCs: endothelial progenitor cells
GMP: good manufacturing practice

## References

[1] Hajishengallis G (2015). Periodontitis: from microbial immune subversion to systemic inflammation. Nat Rev Immunol.

[2] Lamont RJ, Koo H, Hajishengallis G (2018). The oral microbiota: dynamic communities and host interactions. Nat Rev Microbiol.

[3] Hajishengallis G (2000). 2020 New developments in neutrophil biology and periodontitis. Periodontol.

[4] Pan W, Wang Q, Chen Q (2019). The cytokine network involved in the host immune response to periodontitis. Int J Oral Sci.

[5] Elashiry M, Morandini AC, Cornelius Timothius CJ, Ghaly M, Cutler CW (2021). Selective antimicrobial therapies for periodontitis: win the "BATTLE and the War". Int J Mol Sci.

[6] Johnstone RM, Adam M, Hammond JR, Orr L, Turbide C (1987). Vesicle formation during reticulocyte maturation. Association of plasma membrane activities with released vesicles (exosomes). J Biol Chem..

[7] Kibria G, Ramos EK, Wan Y, Gius DR, Liu H (2018). exosomes as a drug delivery system in cancer therapy: potential and challenges. Mol Pharm.

[8] Ahmed F, Tamma M, Pathigadapa U, Reddanna P, Yenuganti VR (2022). Drug loading and functional efficacy of cow, buffalo, and goat milk-derived exosomes: a comparative study. Mol Pharm.

[9] Akbar A, Malekian F, Baghban N, Kodam SP, Ullah M (2022). Methodologies to isolate and purify clinical grade extracellular vesicles for medical applications. Cells.

[10] Kanlikilicer P (2022). Exosome-related methods and potential use as vaccines. Methods Mol Biol.

[11] Kalluri R, LeBleu VS (2020). The biology, function, and biomedical applications of exosomes. Science.

[12] Trubiani O, Marconi GD, Pierdomenico SD, Piattelli A, Diomede F, Pizzicannella J (2019). Human oral stem cells, biomaterials and extracellular vesicles: a promising tool in bone tissue repair. Int J Mol Sci.

[13] Wang X, He L, Huang X, Zhang S, Cao W, Che F (2021). Recent progress of exosomes in multiple myeloma: pathogenesis, diagnosis prognosis and therapeutic strategies. Cancers (Basel).

[14] Gurung S, Perocheau D, Touramanidou L, Baruteau J (2021). The exosome journey: from biogenesis to uptake and intracellular signalling. Cell Commun Signal.

[15] Hessvik NP, Llorente A (2018). Current knowledge on exosome biogenesis and release. Cell Mol Life Sci.

[16] Mobius W, Ohno-Iwashita Y, van Donselaar EG, Oorschot VM, Shimada Y, Fujimoto T (2002). Immunoelectron microscopic localization of cholesterol using biotinylated and non-cytolytic perfringolysin O. J Histochem Cytochem.

[17] Wollert T, Hurley JH (2010). Molecular mechanism of multivesicular body biogenesis by ESCRT complexes. Nature.

[18] Zhang Y, Bi J, Huang J, Tang Y, Du S, Li P (2020). Exosome: a review of its classification, isolation techniques, storage, diagnostic and targeted therapy applications. Int J Nanomedicine.

[19] Trajkovic K, Hsu C, Chiantia S, Rajendran L, Wenzel D, Wieland F (2008). Ceramide triggers budding of exosome vesicles into multivesicular endosomes. Science.

[20] Wei D, Zhan W, Gao Y, Huang L, Gong R, Wang W (2021). RAB31 marks and controls an ESCRT-independent exosome pathway. Cell Res.

[21] de Gassart A, Geminard C, Fevrier B, Raposo G, Vidal M (2003). Lipid raft-associated protein sorting in exosomes. Blood.

[22] Rana S, Zoller M (2011). Exosome target cell selection and the importance of exosomal tetraspanins: a hypothesis. Biochem Soc Trans.

[23] Booth AM, Fang Y, Fallon JK, Yang JM, Hildreth JE, Gould SJ (2006). Exosomes and HIV Gag bud from endosome-like domains of the T cell plasma membrane. J Cell Biol.

[24] Wei H, Chen Q, Lin L, Sha C, Li T, Liu Y (2021). Regulation of exosome production and cargo sorting. Int J Biol Sci.

[25] Yokoi A, Ochiya T (2021). Exosomes and extracellular vesicles: rethinking the essential values in cancer biology. Semin Cancer Biol.

[26] Gurunathan S, Kang MH, Kim JH (2021). A comprehensive review on factors influences biogenesis, functions, therapeutic and clinical implications of exosomes. Int J Nanomedicine.

[27] Busatto S, Pham A, Suh A, Shapiro S, Wolfram J (2019). Organotropic drug delivery: Synthetic nanoparticles and extracellular vesicles. Biomedical Microdevices.

[28] Théry C, Zitvogel L, Amigorena S (2002). Exosomes: composition, biogenesis and function. Nat Rev Immunol.

[29] Tan L, Wu H, Liu Y, Zhao M, Li D, Lu Q (2016). Recent advances of exosomes in immune modulation and autoimmune diseases. Autoimmunity.

[30] Meldolesi J (2018). Exosomes and ectosomes in intercellular communication. Curr Biol.

[31] Donoso-Quezada J, Ayala-Mar S, Gonzalez-Valdez J (2021). The role of lipids in exosome biology and intercellular communication: Function, analytics and applications. Traffic.

[32] Ludwig N, Whiteside TL, Reichert TE (2019). Challenges in exosome isolation and analysis in health and disease. Int J Mol Sci.

[33] Yang D, Zhang W, Zhang H, Zhang F, Chen L, Ma L (2020). Progress, opportunity, and perspective on exosome isolation - efforts for efficient exosome-based theranostics. Theranostics.

[34] Wang J, Ma P, Kim DH, Liu BF, Demirci U (2021). Towards microfluidic-based exosome isolation and detection for tumor therapy. Nano Today.

[35] Sidhom K, Obi PO, Saleem A (2020). A review of exosomal isolation methods: is size exclusion chromatography the best option?. Int J Mol Sci.

[36] Singh K, Nalabotala R, Koo KM, Bose S, Nayak R, Shiddiky MJA (2021). Separation of distinct exosome subpopulations: isolation and characterization approaches and their associated challenges. Analyst.

[37] Zhu L, Sun HT, Wang S, Huang SL, Zheng Y, Wang CQ (2020). Isolation and characterization of exosomes for cancer research. J Hematol Oncol.

[38] Papapanou PN, Sanz M, Buduneli N, Dietrich T, Feres M, Fine DH (2018). Periodontitis: consensus report of workgroup 2 of the 2017 world workshop on the classification of periodontal and peri-implant diseases and conditions. J Periodontol.

[39] Peng Q, Yang JY, Zhou G (2020). Emerging functions and clinical applications of exosomes in human oral diseases. Cell Biosci.

[40] Nik Mohamed Kamal NNS, Awang RAR, Mohamad S, Shahidan WNS (2020). Plasma- and saliva exosome profile reveals a distinct MicroRNA signature in chronic periodontitis. Front Physiol.

[41] Kwon EJ, Kim HJ, Woo BH, Joo JY, Kim YH, Park HR (2022). Profiling of plasma-derived exosomal RNA expression in patients with periodontitis: a pilot study. Oral Dis.

[42] Yu J, Lin Y, Xiong X, Li K, Yao Z, Dong H (2019). Detection of exosomal PD-L1 RNA in saliva of patients with periodontitis. Front Genet.

[43] Huang X, Hu X, Zhao M, Zhang Q (2020). Analysis of salivary exosomal proteins in young adults with severe periodontitis. Oral Dis.

[44] Tobon-Arroyave SI, Celis-Mejia N, Cordoba-Hidalgo MP, Isaza-Guzman DM (2019). Decreased salivary concentration of CD9 and CD81 exosome-related tetraspanins may be associated with the periodontal clinical status. J Clin Periodontol.

[45] Nik Mohamed Kamal NNS, Shahidan WNS (2021). Salivary exosomes: from waste to promising periodontitis treatment. Front Physiol.

[46] Zhang Y, Chen J, Fu H, Kuang S, He F, Zhang M (2021). Exosomes derived from 3D-cultured MSCs improve therapeutic effects in periodontitis and experimental colitis and restore the Th17 cell/Treg balance in inflamed periodontium. Int J Oral Sci.

[47] Shen Z, Kuang S, Zhang Y, Yang M, Qin W, Shi X (2020). Chitosan hydrogel incorporated with dental pulp stem cell-derived exosomes alleviates periodontitis in mice via a macrophage-dependent mechanism. Bioact Mater.

[48] Shimizu Y, Takeda-Kawaguchi T, Kuroda I, Hotta Y, Kawasaki H, Hariyama T (2022). Exosomes from dental pulp cells attenuate bone loss in mouse experimental periodontitis. J Periodontal Res.

[49] Lei F, Li M, Lin T, Zhou H, Wang F, Su X (2022). Treatment of inflammatory bone loss in periodontitis by stem cell-derived exosomes. Acta Biomater.

[50] Nakao Y, Fukuda T, Zhang Q, Sanui T, Shinjo T, Kou X (2021). Exosomes from TNF-alpha-treated human gingiva-derived MSCs enhance M2 macrophage polarization and inhibit periodontal bone loss. Acta Biomater.

[51] Wei J, Song Y, Du Z, Yu F, Zhang Y, Jiang N (2020). Exosomes derived from human exfoliated deciduous teeth ameliorate adult bone loss in mice through promoting osteogenesis. J Mol Histol.

[52] Wu J, Chen L, Wang R, Song Z, Shen Z, Zhao Y (2019). Exosomes secreted by stem cells from human exfoliated deciduous teeth promote alveolar bone defect repair through the regulation of angiogenesis and osteogenesis. ACS Biomater Sci Eng.

[53] Chen X, Wan Z, Yang L, Song S, Fu Z, Tang K (2022). Exosomes derived from reparative M2-like macrophages prevent bone loss in murine periodontitis models via IL-10 mRNA. J Nanobiotechnology.

[54] Mohammed E, Khalil E, Sabry D (2018). Effect of adipose-derived stem cells and their Exo as adjunctive therapy to nonsurgical periodontal treatment: a histologic and histomorphometric study in rats. Biomolecules.

[55] Byun JS, Lee HY, Tian J, Moon JS, Choi J, Lee SH (2021). Effect of salivary exosomal miR-25-3p on periodontitis with insulin resistance. Front Immunol.

[56] Taghavi-Farahabadi M, Mahmoudi M, Rezaei N, Hashemi SM (2021). Wharton's jelly mesenchymal stem cells exosomes and conditioned media increased neutrophil lifespan and phagocytosis capacity. Immunol Invest.

[57] Zhang B, Lai RC, Sim WK, Choo ABH, Lane EB, Lim SK (2021). Topical application of mesenchymal stem cell exosomes alleviates the imiquimod induced psoriasis-like inflammation. Int J Mol Sci.

[58] Mahmoudi M, Taghavi-Farahabadi M, Rezaei N, Hashemi SM (2019). Comparison of the effects of adipose tissue mesenchymal stromal cell-derived exosomes with conditioned media on neutrophil function and apoptosis. Int Immunopharmacol.

[59] Murao A, Tan C, Jha A, Wang P, Aziz M (2021). Exosome-mediated eCIRP release from macrophages to induce inflammation in sepsis. Front Pharmacol.

[60] Zhao J, Li X, Hu J, Chen F, Qiao S, Sun X (2019). Mesenchymal stromal cell-derived exosomes attenuate myocardial ischaemia-reperfusion injury through miR-182-regulated macrophage polarization. Cardiovasc Res.

[61] Shi Y, Kang X, Wang Y, Bian X, He G, Zhou M (2020). Exosomes derived from bone marrow stromal cells (BMSCs) enhance tendon-bone healing by regulating macrophage polarization. Med Sci Monit.

[62] Deng H, Wu L, Liu M, Zhu L, Chen Y, Zhou H (2020). Bone marrow mesenchymal stem cell-derived exosomes attenuate LPS-induced ARDS by modulating macrophage polarization through inhibiting glycolysis in macrophages. Shock.

[63] Ning H, Chen H, Deng J, Xiao C, Xu M, Shan L (2021). Exosomes secreted by FNDC5-BMMSCs protect myocardial infarction by anti-inflammation and macrophage polarization via NF-kappaB signaling pathway and Nrf2/HO-1 axis. Stem Cell Res Ther.

[64] Xin L, Lin X, Zhou F, Li C, Wang X, Yu H (2020). A scaffold laden with mesenchymal stem cell-derived exosomes for promoting endometrium regeneration and fertility restoration through macrophage immunomodulation. Acta Biomater.

[65] Dong B, Wang C, Zhang J, Zhang J, Gu Y, Guo X (2021). Exosomes from human umbilical cord mesenchymal stem cells attenuate the inflammation of severe steroid-resistant asthma by reshaping macrophage polarization. Stem Cell Res Ther.

[66] Heo JS, Lim JY, Yoon DW, Pyo S, Kim J (2020). Exosome and melatonin additively attenuates inflammation by transferring miR-34a, miR-124, and miR-135b. Biomed Res Int.

[67] Zhao H, Shang Q, Pan Z, Bai Y, Li Z, Zhang H (2018). Exosomes from adipose-derived stem cells attenuate adipose inflammation and obesity through polarizing M2 macrophages and Beiging in white adipose tissue. Diabetes.

[68] Shen K, Jia Y, Wang X, Zhang J, Liu K, Wang J (2021). Exosomes from adipose-derived stem cells alleviate the inflammation and oxidative stress via regulating Nrf2/HO-1 axis in macrophages. Free Radic Biol Med.

[69] Wang R, Ji Q, Meng C, Liu H, Fan C, Lipkind S (2020). Role of gingival mesenchymal stem cell exosomes in macrophage polarization under inflammatory conditions. Int Immunopharmacol.

[70] Shahir M, Mahmoud Hashemi S, Asadirad A, Varahram M, Kazempour-Dizaji M, Folkerts G (2020). Effect of mesenchymal stem cell-derived exosomes on the induction of mouse tolerogenic dendritic cells. J Cell Physiol.

[71] Zhang Y, Yan J, Li Z, Zheng J, Sun Q (2022). Exosomes derived from human umbilical cord mesenchymal stem cells alleviate psoriasis-like skin inflammation. J Interferon Cytokine Res.

[72] Elashiry M, Elashiry MM, Elsayed R, Rajendran M, Auersvald C, Zeitoun R (2020). Dendritic cell derived exosomes loaded with immunoregulatory cargo reprogram local immune responses and inhibit degenerative bone disease in vivo. J Extracell Vesicles.

[73] Brown M, Johnson LA, Leone DA, Majek P, Vaahtomeri K, Senfter D (2018). Lymphatic exosomes promote dendritic cell migration along guidance cues. J Cell Biol.

[74] Cosenza S, Toupet K, Maumus M, Luz-Crawford P, Blanc-Brude O, Jorgensen C (2018). Mesenchymal stem cells-derived exosomes are more immunosuppressive than microparticles in inflammatory arthritis. Theranostics.

[75] Du YM, Zhuansun YX, Chen R, Lin L, Lin Y, Li JG (2018). Mesenchymal stem cell exosomes promote immunosuppression of regulatory T cells in asthma. Exp Cell Res.

[76] Zhou J, Lu Y, Wu W, Feng Y (2021). HMSC-derived exosome inhibited Th2 cell differentiation via regulating miR-146a-5p/SERPINB2 pathway. J Immunol Res.

[77] Zheng Y, Dong C, Yang J, Jin Y, Zheng W, Zhou Q (2019). Exosomal microRNA-155-5p from PDLSCs regulated Th17/Treg balance by targeting sirtuin-1 in chronic periodontitis. J Cell Physiol.

[78] Xu L, Geng T, Zang G, Bo L, Liang Y, Zhou H (2020). Exosome derived from CD137-modified endothelial cells regulates the Th17 responses in atherosclerosis. J Cell Mol Med.

[79] Xiao F, Zuo B, Tao B, Wang C, Li Y, Peng J (2021). Exosomes derived from cyclic mechanical stretch-exposed bone marrow mesenchymal stem cells inhibit RANKL-induced osteoclastogenesis through the NF-kappaB signaling pathway. Ann Transl Med.

[80] Zhang L, Wang Q, Su H, Cheng J (2021). Exosomes from adipose derived mesenchymal stem cells alleviate diabetic osteoporosis in rats through suppressing NLRP3 inflammasome activation in osteoclasts. J Biosci Bioeng.

[81] Ren L, Song ZJ, Cai QW, Chen RX, Zou Y, Fu Q (2019). Adipose mesenchymal stem cell-derived exosomes ameliorate hypoxia/serum deprivation-induced osteocyte apoptosis and osteocyte-mediated osteoclastogenesis in vitro. Biochem Biophys Res Commun.

[82] Zhang L, Wang Q, Su H, Cheng J (2022). Exosomes from adipose tissues derived mesenchymal stem cells overexpressing MicroRNA-146a alleviate diabetic osteoporosis in rats. Cell Mol Bioeng.

[83] Wang Q, Shen X, Chen Y, Chen J, Li Y (2021). Osteoblasts-derived exosomes regulate osteoclast differentiation through miR-503-3p/Hpse axis. Acta Histochem.

[84] Cui Y, Fu S, Sun D, Xing J, Hou T, Wu X (2019). EPC-derived exosomes promote osteoclastogenesis through LncRNA-MALAT1. J Cell Mol Med.

[85] Barile L, Vassalli G (2017). Exosomes: therapy delivery tools and biomarkers of diseases. Pharmacol Ther.

[86] Gugliandolo A, Fonticoli L, Trubiani O, Rajan TS, Marconi GD, Bramanti P (2021). Oral bone tissue regeneration: mesenchymal stem cells, secretome, and biomaterials. Int J Mol Sci.

